# Development of a regulatory testing procedure to study the metabolism of pesticides in farmed fish

**DOI:** 10.1002/ps.4007

**Published:** 2015-04-17

**Authors:** Christian Schlechtriem, Ina Bischof, Cornelia Atorf, Elena Bergendahl, Paul Seymour, Paul Whalley

**Affiliations:** ^1^ Fraunhofer Institute for Molecular Biology and Applied Ecology (IME)SchmallenbergGermany; ^2^ SyngentaJealott's Hill International Research CentreBracknellBerksRG42 6EYUK

**Keywords:** pesticide regulation, aquaculture, feed residues, rainbow trout, common carp

## Abstract

**BACKGROUND:**

Diets used in commercial fish farming use significant proportions of crop‐derived commodities, and it is important to understand the potential for transfer of any pesticide residues on the crop into edible tissues in fish. It is a current requirement in the EU that fish metabolism studies must be performed when a pesticide is used in crops where commodities or processed fractions are fed to farmed fish. Fish metabolism studies in both rainbow trout and common carp have been carried out, following the new working document on the nature of pesticide residues in fish using ^14^C‐labelled pesticide.

**RESULTS:**

The ingestion of experimental diets by rainbow trout and common carp resulted in the uptake and metabolism of the test item, as shown by liquid scintillation counting combined with radio‐thin‐layer chromatography. The metabolite profiles for trout and carp were qualitatively similar regarding the main residue. However, species‐specific differences were found regarding the remaining residue with rainbow trout showing additional metabolites in comparison to carp.

**CONCLUSIONS:**

Metabolism studies for regulatory purposes can be carried out with both fish species under laboratory conditions. The experimental design reported is suitable for quantifying the transfer of residues to edible tissues and enables characterisation of the chemical nature of residues. © 2015 Fraunhofer Institute for Molecular Biology and Applied Ecology (IME). Pest Management Science published by John Wiley & Sons Ltd on behalf of Society of Chemical Industry.

## 1 INTRODUCTION

Today, aquaculture production provides roughly 50% of the fish consumed worldwide.^1^ Owing to the increase in fish farming and the increase in use of plant commodities as a source of feed, there is a need to estimate the levels and nature of pesticide residues in edible products in fish. Therefore, the EU published new data requirements for fish as part of the approval process for pesticides.^2^, ^3^ Fish metabolism data provide an estimate of total terminal residues and characterise the chemical nature of residues that may occur in edible commodities of fish exposed to pesticides. Currently, metabolism studies with fish are required when pesticides of log *K*_ow_ > 3 are used in crops fed to farmed fish, which may lead to significant residues in fish feed, considered to be >0.1 mg kg^−1^ total diet (dry weight basis).^3^ Guidance documents on metabolism studies for ruminants, poultry and pigs are available,^4^, ^5^ but these are not fully applicable to fish because of differences in the testing environment and husbandry practices. In particular, radiolabelled test material is difficult to apply under aquatic conditions, and experimental diets enriched with moderately lipophilic (log *K*_ow_ < 5) test material need to be stabilised, to avoid leaching of the test compound from the fish‐feed pellets, prior to ingestion by the experimental animals.^6^ In addition, large volumes of contaminated water may result from metabolism studies and need to be treated using powerful filter technology.

In contrast to the existing regulatory testing procedure for biomagnification, according to OECD TG 305,^7^ fish metabolism studies reflecting oral ingestion need to be carried out with larger animals of marketable size. This is to provide sufficient tissue for the quantitation of residues in the fish product (fillet, liver), and to enable identification of radiolabelled metabolites. The use of small groups of larger fish requires the availability of large tank facilities in order to maintain a maxium loading rate of 1 g fish (wet weight) L^−1^ water day^−1^. A working document on the nature of pesticide residues in fish was recently published to provide guidance on the performance of fish metabolism studies.^8^ Important inland aquaculture species reared for human consumption such as rainbow trout (*Oncorhynchus mykiss*) or common carp (*Cyprinus carpio*) are the recommended species. Both species are easily reared under laboratory conditions but are characterised by different feeding behaviours (carnivorous versus omnivorous).

The objective of this study was to assess the practicality of conducting metabolism studies in fish for regulatory purposes. A test system using flow‐through conditions and incorporating rainbow trout and common carp (>250 g) as test species has been developed and is reported.

## 2 MATERIALS AND METHODS

### 2.1 Standards and reagents

The test item was a pesticide with a log *K*_ow_ of 3.3 and a water solubility of ca 70 mg L^−1^ (25 °C). The specific activity of the radiochemical was 5.028 MBq mg^−1^ (trout study) and 5.169 MBq mg^−1^ (carp study), with a purity of >98%. The radiolabelled test item and non‐labelled reference compounds (parent and two known metabolites) were provided by Syngenta UK (Bracknell, UK). All solvents used were high‐performance liquid chromatography grade and purchased from J.T. Baker, Deventer, the Netherlands, if not stated otherwise.

### 2.2 Feed preparation

Commercially available non‐medicated feed for trout (Milkivit‐type F‐2P B40; Trouw Nutrition, Burgheim, Germany) and common carp (Milkivit‐type Pro Aqua K18 C5; Trouw Nutrition) with a pellet size of 4 and 5 mm respectively, and appropriate to the species under investigation, was used for this study. A feed preparation protocol, including a solvent spiking procedure followed by pellet coating ensuring the production of stable and homogeneously fortified test diets, was applied.^6^ In brief, pellets were spiked in a rotary evaporator with the [^14^C]‐labelled test item to reach a nominal concentration of 12 mg kg^−1^ (equivalent to approximately 60 MBq kg^−1^). The target concentration was selected to ensure a dose of 10 mg kg^−1^, the minimum concentration recommended in regulatory livestock metabolism studies. The spiked pellets were surface coated with calcium alginate to avoid leaching of the test item prior to ingestion by the fish. The coating was carried out according to an established protocol;^9^ however, only one‐quarter of the recommended amount of alginate solution was applied to improve the stability of the pellets.^6^ The pellets were stored at −20 °C. A triplicate sample of each batch of spiked feed was taken for analysis of radiochemical content.

### 2.3 Test species

Fertilised rainbow trout (*Oncorhynchus mykiss*) eggs were obtained from Sauerländer Forellenzucht Thomas Rameil (Lennestadt, Germany). The animals were reared in the Fraunhofer‐IME hatchery, Schmallenberg, to the experimental size of around 300 g. Common carp (*Cyprinus carpio*) weighing ca 200–300 g were provided by a commercial hatchery (Vollmann‐Schipper, Mindelaltheim, Germany). The health status of the fish and their suitability for inclusion in the study was assessed after an acclimatisation period lasting 4 weeks. Finally, five rainbow trout and common carp with an average weight of 307.6 g (±26.5 SD) and 244.2 g (±19.1 SD), respectively, were selected 7 days before the onset of the study and transferred to the experimental tank. The animals were fed on commercial diet until the start of the experiment.

### 2.4 Experimental conditions

**Figure 1:**
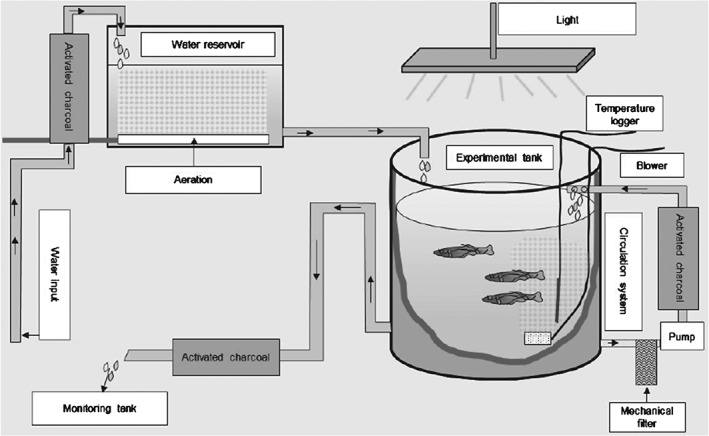
Schematic of the experimental set‐up.

The fish metabolism experiments were carried out under flow‐through conditions (45 L h^−1^) in a large experimental tank with a volume of 2 m^3^ filled with 1 m^3^ of preconditioned tap water (Fig. 1). The water purification included filtration with activated charcoal, aeration and passage through a limestone (calcium carbonate) column. Water in the experimental tank was constantly recirculated through an additional filter column filled with activated charcoal (∼700 g) to avoid the accumulation of the radiolabelled test item and metabolites dissolved in the water of the experimental tank. The effluent of the experimental tank was passed through a filter column filled with activated charcoal (∼700 g) and finally collected in a separate tank (2 m^3^) to monitor the remaining total radioactivity.

The temperature of the water in the experimental tank was maintained in the range 13.8–14.5 °C for rainbow trout and 20.7–22.7 °C for common carp and was recorded daily during the acclimatisation and dosing period. The oxygen saturation and pH were monitored and kept constantly above 80% and between 8.0 and 8.2 respectively. The light regime was adjusted to a 16:8 h dark:light cycle.

### 2.5 Feeding of animals

Fish were fed at a daily feeding rate of 3% of their average live weight in compliance with the recommendations given by the feed manufacturer. The average live weight of the experimental animals was estimated 1 week before and at the onset (only carp) of the experiment. The exact feeding rate applied during the experiment was recorded to estimate the total amount of test item administered to the experimental tank. The daily ration was distributed on the surface of the water once daily for 10 consecutive days. Generally, feeding was carried out in the morning until feed uptake by the fish visibly slowed down. The remaining part of the feed portion was then administered in smaller parts until early noon of the same day. As far as possible, it was ensured that the food ration was ingested immediately by the fish to minimise losses of test radiochemical by leaching into water.

### 2.6 Calculation of the specific growth rate and feed conversion ratio

The specific growth rate (SGR) was calculated on the basis of the average weight of fish collected from the experimental tank, using the following formula:

$$SGR=ln\left(W_{t_{2}}\right)-ln\left(W_{t_{1}}\right)/\left(t_{2}-t_{1}\right)*100$$

where $W_{t_{2}}$ is the weight at the end of the experimental period, $W_{t_{1}}$ is the weight at the start of experimental period, t_2_ is the end of the experimental period, and t_1_ is the start of the experimental period.

The feed conversion ratio (FCR) was calculated as a measure for the conversion of feed to body weight gain, using the following formula:

$$FCR=\frac{\text{mass of feed consumed}\;\left(\text{dry}\right)}{\text{increase}\;\mathrm{in}\;\text{mass of animal produced}\;\left(\text{wet}\right)}$$

### 2.7 Sample collection and preservation

Triplicate water samples were taken from the experimental tank around 2 h before and after feeding every day throughout the experiment to estimate the total radioactivity in the water. Additonal water samples were taken from the effluent tank before draining to allow the calculation of a mass balance. Faeces were siphoned daily within 2 h of feeding and collected for analysis of radiochemical content. On day 10, 6 h after the last feeding event, the five fish were caught individually with a dip net, anaesthetised with MS‐222 and killed by cutting the spinal cord with a scalpel. The sacrificed animals were weighed immediately and washed with water, and the liver tissue, stomach and gut were removed. Dissected guts and stomachs (only trout) were emptied and the contents (GI tract content) were removed and collected. Muscle samples were taken from the disembowelled fish, and the skin was removed from the fillets. The weight of each tissue was recorded in the raw data. All samples were stored frozen (ca −20 °C) until taken for analysis.

At the end of the study, the activated charcoal was removed from the filter columns and thoroughly mixed with the collected faeces and GI tract contents. Aliquots were taken for analysis.

### 2.8 Sample analysis

Prior to the start of the experiments, the concentration of radiochemical in the experimental diets was determined. In order to determine the homogeneity of the radioactive test item on the fish feed pellets, triplicate samples (∼3 g) were prepared and analysed by liquid scintillation counting (LSC) (Packard Tri‐Carb; Packard, Downers Grove, IL, USA) following a similar protocol to that used for the analysis of the collected fish tissues described below.

The tissue samples dissected from the experimental animals were homogenised by means of a Thermomix (Vorwerk, Wuppertal, Germany) and ULTRA‐TURRAX^®^ (IKA, Staufen, Germany). Skin was ground to a fine powder while frozen with the aid of liquid nitrogen. Homogenised samples were kept frozen at −20 °C until analysis.

Aliquots of each sample were analysed for radioactivity. Homogenised material (maximum 250 mg) was added to LSC vials and mixed with 4 mL of Soluene^®^‐350 (Packard). Samples were placed in a water bath overnight at 50 °C and agitated slowly. Finally, 1 mL of methanol was added to aid miscibility. Prior to the quantification by LSC, 15 mL of Hionic‐Fluor™ scintillation cocktail (Packard) was added to each sample. Each sample was measured for 5 min against a blank treated in the same way as the samples. Triplicate samples were analysed in order to ensure reproducibility.

Around 700 g of filter material (activated charcoal) was collected from each filter column. Filter material, mixed with faeces and GI tract contents, was dried at 92 °C. Samples were weighed and homogenised prior to combustion (OX500 biological oxidiser; R.J. Harvey Instrument Corp., Tappan, NY). LSC measurements were performed by means of a Packard Tri‐Carb liquid scintillation analyser.

Water samples (5 mL) were mixed immediately with 15 mL Ultima Gold™ LLT (Perkin Elmer, Groningen, The Netherlands) and analysed by LSC. Each sample was measured for 5 min against a blank. Triplicate samples were analysed in order to ensure reproducibility.

### 2.9 Mass balance

Based on the results obtained from the sample analysis, mass balances for the radiochemical administered to the test systems during the experiments were calculated. Only a limited part of the muscle tissue was sampled from rainbow trout for chemical analysis. The distribution of the accumulated radiochemical between the different tissues was therefore determined assuming an average proportion of muscle tissue of 43%, which was previously estimated for control animals of a comparable size. The amount of radiochemical in the different matrices was calculated. The total radioactivity measured in the test system was compared with the total amount of radiochemical (kBq) applied during the fish metabolism study, and the recovery of the applied dose (%) was determined.

### 2.10 Extraction of radioactive residues in fillet and liver

Approximately 4.5 g of trout and carp fillet and liver/hepatopancreatic samples were accurately weighed and homogenised with an Ultra Turrax^®^ in 3 mL of acetonitrile. The dispersing probe was rinsed with 2 × 1 mL of acetonitrile, and 3 mL of n‐hexane was added to the homogenate. Samples were shaken for 20 min at 140 rpm and then placed in an ultrasonic bath for 5 min, followed by 20 min of centrifugation at 2500 rpm. The supernatant of each sample was collected, and two more extractions were performed as described above. The solvent fractions were pooled and analysed for total radioactivity by liquid scintillation counting (Tri‐Carb; Packard). Two 250 μL samples were taken from each solvent fraction, and each mixed with 4 mL of Ultima Gold™ Cocktail. The extraction residue/pellet was dried overnight at room temperature, and aliquots were solubilised with tissue solubiliser (Soluene^®^‐350) for radio assay. The radioactivity in the samples was determined by liquid scintillation counting (Tri‐Carb; Packard) in order to calculate the total amount of non‐extractable residues in the tissue samples. Acetonitrile extracted 75.3% of the ^14^C‐labelled material from carp hepatopancreatic tissue, 77.0% from trout fillet, and 87.9% from carp fillet. Hexane extracted 5.3% of the ^14^C from carp hepatopancreatic tissue, 5.1% from trout fillet and 10.5% from carp fillet. Non‐extractable residues were 19.4% for carp hepatopancreatic tissue, 17.9% for trout fillet and 1.6% for carp fillet.

### 2.11 Thin‐layer chromatography

Metabolic profiles of the test compound in fillet and liver/hepatopancreatic tissue of trout and carp were determined. Aliquots of acetonitrile extracts (approximately 5 Bq) were applied to a normal‐phase silica gel 60 thin‐layer chromatography (TLC) plate with fluorescence indicator (Merck TLC Silicagel 60WF254s, 20 × 20), along with non‐labelled parent reference compound and two reference metabolites (each approximately 20 µg). The TLC plates were developed in ethyl acetate/2‐propanol/water (65:25:15 by volume) and then dried and analysed by a BAS‐1000 Bio‐Imaging Analyser (Fujifilm, Tokyo, Japan) and AIDA software (Raytest, Straubenhardt, Germany). Non‐labelled standards were visualised by UV light.

## 3 RESULTS

### 3.1 Feed preparation

Two batches of homogeneously spiked feed were prepared by spray application. The rainbow trout diet contained 10.6 mg kg^−1^, equivalent to ca 53 MBq, and the carp diet contained 10.6 mg kg^−1^, equivalent to ca 55 MBq. The spiked diet was palatable to the fish and accepted readily during the study. Pellets were ingested by both species within 1–2 min of feeding.

### 3.2 Growth performance and feed conversion

All experimental animals survived and showed no change in behaviour in response to the administration of the experimental diet. Rainbow trout demonstrated good growth throughout the study, with an average weight gain of around 125 g fish^−1^ during the acclimatisation and exposure period. The specific growth rate of the animals was 2.0% day^−1^. In comparison, common carp showed a lower growth rate (1.1% day^−1^), leading to an average weight gain of 52 g fish^−1^. The feed conversion (FCR) was acceptable in the study on rainbow trout (1.2); however, the increased FCR estimate observed for common carp (2.4) clearly points to a reduced digestibility of the experimental diet (Table 1).

**Table 1:** Feed conversion ratio (FCR) and specific growth rate (SGR) of experimental animals during the exposure period

	Rainbow trout	Common carp
Specific growth rate^a^		
$W_{t_{0}}$ ± SD (g)	308 ± 27	244 ± 19
$W_{t_{1}}$ ± SD (g)	—b	267 ± 19
$W_{t_{2}}$ ± SD (g)	433 ± 54	297 ± 18
t_1_ − t_0_ (days)^c^	7	7
t_2_ − t_1_ (days)^d^	10	10
${SGR}_{t_{0}-t_{1}}$ (% day^−1^)		1.3
${SGR}_{t_{1}-t_{2}}$ (% day^−1^)		1.1
${SGR}_{t_{0}-t_{2}}$ (% day^−1^)	2.0	1.1
Feed conversion ratio		
$\text{Average body weight}\;{\text{gain}}_{t_{0}-t_{2}}$ (g fish^−1^)	125	52
$\text{Average feed}\;{\text{intake}}_{t_{0}-t_{2}}$ (g fish^−1^)^e^	156	124
${FCR}_{t_{0}-t_{1}}$		2.0
${FCR}_{t_{1}-t_{2}}$		2.7
${FCR}_{t_{0}-t_{2}}$	1.2	2.4

a $W_{t_{1}}$ = average body weight at t_1_; $W_{t_{2}}$ = average body weight at t_2_; $W_{t_{0}}$ = stocking weight of fish; SD = relative standard deviation.

b No weight measurement at start of exposure period to avoid stress.

c t_0_ = onset of acclimatisation period; t_1_ = day 0 of exposure period.

d t_2_ = day 10 of exposure period.

e Daily feeding rate equivalent to 3% of average body weight.

### 3.3 Mass balance

During the metabolism study on rainbow trout, 501 g of spiked feed containing 26.7 MBq was applied to the experimental tank. The total recovery of radioactivity was 95%, with the highest proportion of the radioactivity found in the water (75%) or bound to faeces and gut content pooled with activated charcoal (23%), which was used as filter substrate to treat the effluent of the experimental tank. Around 2% (441 kBq) of the total amount of radioactivity applied was found in the test animals (Table 2). Animals were exposed to a moderate concentration of dissolved radiolabelled compounds in the experimental tank of 1675 Bq L^−1^, on average equivalent to 333 ng L^−1^ related to ^14^C‐labelled test item. A chemical characterisation of dissolved compounds was not carried out.

**Table 2:** Mass balance of total radioactivity (kBq) applied during the study

	Rainbow trout (kBq)	%	Common carp (kBq)	%
Total amount of kBq applied during the fish metabolism study	26 743	100	25 102	100
Water	19 083	(75)^a^	22 797	(79)^a^
Activated carbon/faeces	5996	(23)^a^	5759	(20)^a^
Fish	441	(2)^a^	286	(1)^a^
Recovery	25 520	95	28 842	115

a (%) = percentage of total radioactivity measured (kBq).

Owing to the lower size of the animals, only 402 g of spiked feed containing 25.1 MBq was fed to common carp. The radioactivity was fully recovered (115%) in the different matrices collected during the study with water and activated charcoal (pooled with gut contents) representing 79 and 20% of the dose respectively. The test animals without gut content contained only 1% of the total amount of radioactivity applied (Table 2). Also in this study, moderate concentrations of dissolved radiolabelled compounds were measured in the experimental tank with 1513 Bq L^−1^, on average equivalent to 293 ng L^−1^ related to ^14^C‐labelled test item. The dissolved compounds were not further analysed with respect to their chemical composition.

### 3.4 Tissue distribution

The fillets dissected from rainbow trout and common carp represented on average 43 and 35% of the animals' weight respectively. Liver tissue collected from rainbow trout was smaller (6.35 g) compared with the hepatopancreatic tissue collected from common carp (12.45 g). With 88.24 kBq animal^−1^, the larger rainbow trout (398.8 g animal^−1^) contained a higher amount of total radioactivity than common carp with 57.2 kBq animal^−1^ (284.8 g). Based on the radioactive residues measured in liver and fillet of rainbow trout, the equivalent concentration of the test item in the tissues was 0.16 and 0.013 µg g^−1^ respectively. Fillet and hepatopancreatic tissue dissected from common carp contained 0.026 and 0.07 µg test item equiv g^−1^ respectively (Tables 3 and 4).

**Table 3:** Tissue distribution (rainbow trout)

	Average weight c (g)	%CV	Tissue distribution (%)	Total radioactivity (kBq)	%CV	Total radioactivity (%)	Concentration of test item^d^ (µg g^−1^)	%CV
Total animal^a^	399	11	100	88	18	100	0.044	9
Carcass^b^	227	11	57	77	20	88	0.067	12
Muscle tissue (fillets)	171	11	43	11	25	12	0.013	22
Liver tissue	6	26	2	5	30	6	0.160	44

a Without gut and stomach content.

b All tissues without fillets.

c n = 5.

d Equivalent concentration of test item calculated from total radioactivity in tissues.

**Table 4:** Tissue distribution (common carp)

	Average weight^c^ (g)	%CV	Tissue distribution (%)	Total radioactivity (kBq)	%CV	Total radioactivity (%)	Concentration of test item^d^ (µg g^−1^)	%CV
Total animal^a^	285	7	100	57	33	100	0.039	34
Carcass^b^	187	11	65	44	30	78	0.045	32
Muscle tissue (fillets)	98	2	35	13	50	22	0.026	50
Hepatopancreatic tissue	13	23	4	5	33	9	0.070	41

a Without gut content.

b All tissues without fillets.

c n = 5.

d Equivalent concentration of test item calculated from total radioactivity in tissues.

### 3.5 Determination of metabolic profiles

**Figure 2:**
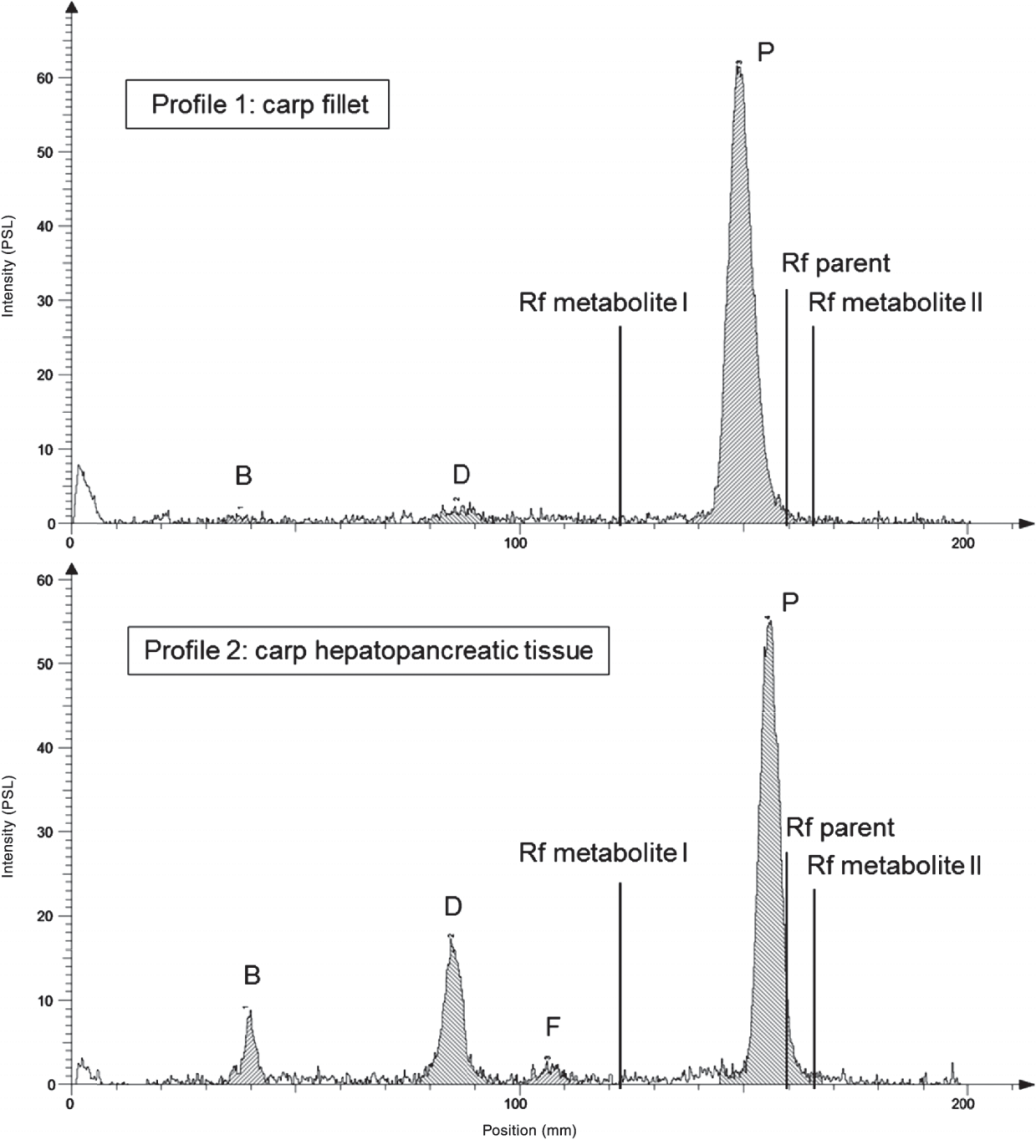
Chromatographic profile of test item metabolism in fillet (profile 1) and hepatopancreatic tissue (profile 2) of common carp.

**Figure 3:**
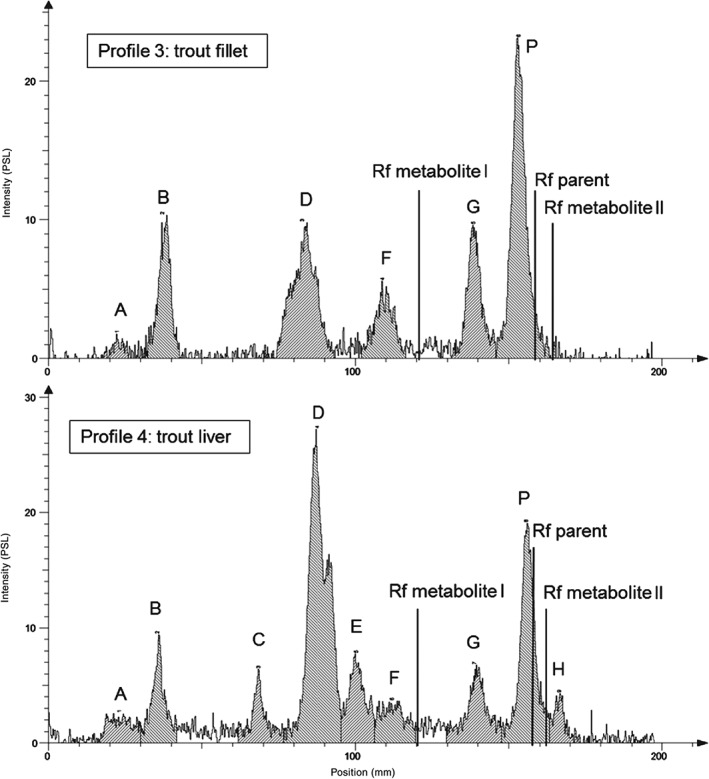
Chromatographic profile of test item metabolism in fillet (profile 3) and liver (profile 4) of rainbow trout.

Metabolic profiles of the test compound in acetonitrile extracts from fillet and liver tissue of trout and carp are presented in Figs 2 and 3. The identification of the metabolites was conducted by Rf value comparison with authentic standards (parent, Rf = 0.89; metabolite I, Rf = 0.65; metabolite II, Rf = 0.92) (Table 5).

**Table 5:** Chromatographic profile of pesticide metabolism in fillet and hepatopancreatic/liver tissue of common carp and rainbow trout

Rf value range	Compound	Integral background (%)			
		Carp fillet	Carp liver	Trout fillet	Trout liver
0.02	Metabolite A	—	—	1.55	4.14
0.11–0.14	Metabolite B	1.60	6.51	13.20	7.61
0.32	Metabolite C	—	—	—	5.50
0.41–0.44	Metabolite D	4.55	20.60	23.70	35.90
0.52	Metabolite E	—	—	—	8.82
0.55–0.61	Metabolite F		3.58	9.85	6.08
0.76–0.77	Metabolite G	—	—	15.00	9.13
0.81–0.88	Parent	93.90	69.40	36.70	19.40
0.95	Metabolite H = reference metabolite II	—	—	—	3.41

Reference metabolite II: Rf value = 0.92; reference metabolite I: Rf value = 0.65, not identified; reference parent compound: Rf value = 0.89.

For carp fillet and hepatopancreatic tissue and trout fillet, the parent compound was detected as the major residue, accounting for 93.9, 69.4 and 36.7% of the total radioactivity residue (TRR) respectively. In trout liver, the parent compound comprised the second highest TRR at 19.4%. In all tissue samples, metabolite D was the prominent metabolite, with TRR of 4.55–35.9%. In the trout liver, eight metabolites (A, B, C, D, E, F, G and H), comprising 80.6% of total peak area, were detected, with six of these found in the fillet (metabolites A, B, D, F, G and H), comprising 63.3% of total peak area. In carp tissue, generally, fewer metabolites were detected. Liver extracts were demonstrated to contain metabolites B, D and F, comprising 30.6% of total peak area, whereas in fillet extracts two metabolites (B and D) were found in significantly lower amounts (6.1% of total peak area). Generally, all metabolites present in fillet tissue were also found in liver tissue, but liver tissue was shown to contain those metabolites in higher amounts and to contain additional metabolites, which were not detected in the fillet. Metabolite H, found solely in trout liver, was tentatively identified as ‘metabolite II’ by comparing the Rf value with the metabolite II standard.

## 4 DISCUSSION

The working document on the nature of residues in fish^8^ recommends rainbow trout and common carp as the test species for fish metabolism studies. This recommendation is based on the importance of these aquaculture species for human consumption. This study showed that fish metabolism studies can be carried out with both species. Following the suggested study design, total terminal residues in edible fish commodities could be estimated and their major components identified. Based on the data generated in fish metabolism studies, a decision on the need for feeding studies to estimate the magnitude of residues in fish can be made. When radioactive residues greater than 0.01 mg kg^−1^ tissue are observed in fish commodities from ingestion of pesticides at levels expected in fish feed (dietary burden), thorough identification of the residues followed by a feeding study is generally necessary.^3^ Therefore, in fish metabolism studies, the daily dosage of fish must at least match the maximum daily exposure based upon the calculation of dietary burden. However, there may be analytical problems if insufficient dose rates of less than 10 mg kg^−1^ dry feed are used. In this study, experimental diets with 10 mg kg^−1^ radiolabelled compound (dry weight basis) were fed, which is equivalent to 100 times the anticipated dietary burden of the test item to fish of 0.1 mg kg^−1^ dry feed. This led to a total radioactivity in edible tissues higher than 0.01 mg kg^−1^ tissue (limit of quantification), which allowed the analysis of labelled components discussed below. With respect to the high dose rates applied in this study, the measured tissue concentrations would be unlikely to require a fish feeding study within a risk assessment.

For fish, the use of feed pellets enriched with radiolabelled test item is recommended. Owing to their nutritionally balanced composition, high palatability and pellet stability, commercial diets should be used. However, test items may dissolve quickly from surface‐spiked test diets in water, especially when compounds of moderate lipophilicity are applied. In this case the coating of surface‐spiked pellets may be required. Experimental diets tested in this study were stabilised by alginate coating, which was shown to reduce significantly the leaching losses of the radiolabelled substance tested in this study.^6^ Pellets were readily ingested by rainbow trout and common carp within 1–2 min of application, confirming the high palatability of the experimental diet. However, in contrast to rainbow trout, a low growth rate and a high feed conversion ratio were observed for common carp. Biopolymers such as alginate may have detrimental effects on nutrient digestibility in fish, as they accelerate gastrointestinal transit time.^10^ A feeding study on rainbow trout, however, showed that animals can easily be adapted to calcium‐alginate‐coated test diets. Growth performance and feed conversion of the animals were acceptable, as confirmed in this study.^11^ In comparison with rainbow trout, common carp showed a lower growth performance and feed conversion, which might be explained by their different digestive system^12^ and a reduced capacity to digest alginate‐coated diets. To ensure a complete ingestion of the daily ration of the experimental diet from the beginning of the experiment, test animals should be adapted to the commercial diet and the coating material during the acclimatisation period. If sufficiently lipophilic test items are applied, the coating of spiked feed pellets should be avoided. Ration size should be calculated on the basis of feeding charts provided by the feed manufacturer, which are designed to induce maximum growth under given temperature conditions. In this study, animals were fed a daily ration equivalent to 3% of the body weight measured at the onset of the experiment. The ration was not adjusted to the increase in body weight during the study, which consequently led to a constant decrease in the dose rate until the end of the study. A weekly adjustment of the daily ration in relation to weight increment should be considered, especially when substances of higher lipophilicity are applied, which require experimental periods longer than 7 days to reach steady‐state conditions. This would require an additional weighing of the animals prior to the end of the feeding period, which may induce stress. Alternatively, the expected weight increment could be predicted on the basis of the specific feed conversion rates available for commercial diets. However, overfeeding must be avoided to ensure the complete ingestion of the experimental diet.

Ideally, dosing should be continued until tissue concentrations reach a steady state. The time to steady state can be predicted according to equations described in OECD TG 305.^7^ However, so far, there is no way to determine whether residues have reached a plateau without serial sacrifice of fish and analytical determination of residue. In practice, dosing should last for no longer than 10–14 consecutive days owing to high experimental inputs.^8^

As described above, high concentrations of the test item (≥10 mg kg^−1^) in the experimental diet may support the identification, characterisation and quantification of radioactive residues. However, care must be taken that the dose applied is still below the toxic concentration of the test item for fish. Prefeeding studies with separate animals are highly recommended to confirm the acceptable dose level prior to the experiment, and to avoid negative effects in response to the administration of the experimental diet. In this study, no changes in behaviour or mortality were observed.

The use of a radiolabelled test item requires the availability of an experimental tank that is equipped with a sufficiently sized filter system to remove the dissolved test item from the water. Owing to the alginate coating of the experimental feed and the immediate uptake of the pellets after feeding, leaching of the test substance into the water prior to ingestion by the fish could be mostly excluded. Therefore, the amount of radioactivity that was measured in the water must have been excreted in urine, via the gills or released from the faeces. The activated‐charcoal‐based filter applied in this study could significantly reduce the amount of radiolabelled compounds in the test water, but the size of the filter was obviously not sufficient to remove the test item and potential metabolites completely. However, test water was constantly replaced during the study. Faeces were constantly removed from the experimental tank to prevent leaching of excreted test item. In this way, the concentration of dissolved radiolabelled compounds could be reduced to avoid a significant impact of bioconcentration processes on the tissue concentrations estimated in this study. Considering the low bioconcentration potential of the test item (BCF = 76; BCFWIN™, US‐EPA) a major accumulation of dissolved radiolabelled compounds in the experimental animals can be excluded. The constant monitoring of the water conditions showed that the experimental system provided optimal culture conditions for both species.

In this study, small groups of fish were treated, which minimises animal usage and is thus in line with the 3R principles. First of all, grouping of fish has practical reasons, because only a single tank has to be managed during the study. Secondly, dosing fish together provides a more attractive, less stressful environment to the fish. The results of this study show that the total radioactivity measured in liver and muscle tissue of individual fish is quite comparable, even if the size of the animals differs significantly. It should be noted that the objective of fish metabolism studies is not to conduct statistical analyses of the radioactive residue in edible matrices but rather to provide a qualitative appreciation of the absorption, translocation and disposition of residues. However, the liver of fish represents a relatively small proportion of the body weight, and therefore the identification of metabolites, even after pooling of five fish livers, may still be a particular challenge. The radioactive residues measured in the fillet and liver of rainbow trout and carp both exceeded the limit of quantification of 0.01 mg kg^−1^, which allows the characterisation of metabolites. The liver tissue dissected from the five rainbow trout provided only a very limited amount of material (∼32 g) for further analysis on biotransformation pathways. In contrast, the collection of hepatopancreatic tissue from common carp resulted in a higher amount of tissue material (∼62 g).

The mass balance of the [^14^C]‐labelled test substance showed that only a minor proportion (approximately 1–2%) accumulated in the sacrificed fish during the course of the study. The highest proportion of test substance (≥75%) was found at low concentrations in the large volume of water released from the experimental tank or bound to activated charcoal. The vast majority of this radioactivity was attributable to urine and very small particles of faeces, but not to leaching from the feed pellets. A total recovery of 95 and 115% of the applied test substance was achieved in the fish studies, which is an acceptable mass balance figure for metabolism studies.

Extracts of homogenised liver and fillet samples collected during the study were analysed by TLC to identify labelled components. In general, the metabolite profiles for trout and carp were qualitatively similar, with consistency for the main residue, as parent and metabolite D. However, species‐specific differences in the chromatographic profile of pesticide metabolites were found regarding the remaining residue with rainbow trout showing additional metabolites (A, C, E, G, H) in comparison to carp. This would suggest that species‐specific differences could exist and therefore further metabolism studies with both species should be carried out with other test items to elucidate whether regulatory relevant species‐specific differences in the metabolite profile may occur.

## 5 CONCLUSIONS

This study demonstrates the feasibility of conducting regulatory metabolism studies in rainbow trout or common carp under laboratory conditions. These studies are suitable for quantifying total residues and characterising the chemical nature of residues that may occur in edible tissues of fish following ingestion of feed containing a pesticide residue.
